# The secreted factor Ag1 missing in higher vertebrates regulates fins regeneration in *Danio rerio*

**DOI:** 10.1038/srep08123

**Published:** 2015-01-29

**Authors:** Anastasiya S. Ivanova, Igor N. Shandarin, Galina V. Ermakova, Andrey A. Minin, Maria B. Tereshina, Andrey G. Zaraisky

**Affiliations:** 1 Shemyakin-Ovchinnikov Institute of Bioorganic Chemistry, Russian Academy of Sciences, Moscow, 117997, Russia; 2 Koltzov Institute of Developmental Biology, Russian Academy of Sciences, Moscow, 119334, Russia

## Abstract

*Agr* family includes three groups of genes, *Ag1*, *Agr2* and *Agr3*, which encode the thioredoxin domain-containing secreted proteins and have been shown recently to participate in regeneration of the amputated body appendages in amphibians. By contrast, higher vertebrates have only *Agr2* and *Agr3*, but lack *Ag1*, and have low ability to regenerate the body appendages. Thus, one may hypothesize that loss of *Ag1* in evolution could be an important event that led to a decline of the regenerative capacity in higher vertebrates. To test this, we have studied now the expression and role of *Ag1* in the regeneration of fins of a representative of another large group of lower vertebrates, the fish *Danio rerio*. As a result, we have demonstrated that amputation of the *Danio* fins, like amputation of the body appendages in amphibians, elicits an increase of *Ag1* expression in cells of the stump. Furthermore, down-regulation of *DAg1* by injections of Vivo-morpholino antisense oligonucleotides resulted in a retardation of the fin regeneration. These data are in a good agreement with the assumption that the loss of *Ag1* in higher vertebrates ancestors could lead to the reduction of the regenerative capacity in their modern descendants.

Agr proteins belong to the superfamily of the thioredoxin domain-containing protein disulphide isomerases, but differ from most of the latter due to their ability to be secreted out of the cell^1^. Because of this property, Agrs can operate as non-cell-autonomous factors, regulating such processes as cell growth and differentiation during the embryonic development, tumorogenesis and regeneration^2^,^3^,^4^,^5^,^6^,^7^,^8^,^9^. In particular, it have been shown recently that all three sub-families of Agrs, Ag1, Agr2 and Agr3, are involved in regulation of the limb and tail regeneration in the *Xenopus laevis* tadpoles (Ivanova et al., 2013). Interestingly, as we have established in this work, higher vertebrates that demonstrate low ability to regenerate the body appendages, including reptiles, birds and mammals, have no genes of *Ag1* subfamily, which were apparently lost by their ancestors. In connection with this, an attractive hypothesis might be formulated, according to which just the extinction of *Ag1* during evolution could be one of the events that resulted in a reduced regenerative capacity of modern higher vertebrates. Accordingly, this hypothesis predicts that *Ag1* could be also involved in the process of appendages regeneration in other lower vertebrates, which, by contrast to higher vertebrates, have this gene and demonstrate high regenerative capacity.

To test this prediction, we have investigated now the involvement of *Ag1* in the process of fins regeneration in zebrafish (*Danio rerio*). By contrast to amphibians, *Agr* family in bony fishes, including *Danio*, is represented by only two genes: *DAg1*, that is lost in higher vertebrates, and *DAgr2*, which is a common ancestor of both *Agr2* and *Agr3* in amphibians and higher vertebrates^5^. The expression pattern of *DAgr2* in Danio embryos and in adults was recently described^10^. As far as we know, however, there is no literature data on *DAg1* expression, neither in embryogenesis, nor during regeneration of the amputated fins. Therefore in the present work, we first of all cloned *DAg1* cDNA and investigated its expression by the whole mount in situ hybridization and qRT-PCR in embryonic development. In addition, we analyzed by the same methods the dynamics of *DAg1* expression during regeneration of the amputated caudal, pectoral and pelvic fins in adult fishes. As a result, we have found out that the expression of *DAg1* is well correlated with the regeneration process. Unlike the Xenopus *Xag1/2*, however, *DAg1* is intensively expressed already in the epithelium of the intact fins, but the level of its expression drops down at the 1 day post amputation (1 dpa). After that, the expression of *DAg1* begins to gradually increase, even exceeding by 5 dpa the initial level in the intact fins. Given that the expression of another fish's representative of *Agr* family, *DAgr2*, was not yet analyzed during fins regeneration, we performed in addition in the present work such an analysis, which yielded results very resembling those obtained for *DAg1*. Finally, by down-regulating *DAg1* functioning by Vivo-morpholino antisense oligonucleotides, we demonstrate that this protein is essential for the fin regeneration. Thus, our data demonstrate tight involvement of both *Agr* genes in the process of fins regeneration, which in turn is in a good agreement with the hypothesis implying that the loss of Ag1 in higher vertebrates' ancestors could be a cause of the reduction of regenerative capacity in their modern descendants.

## Results

### *DAg1* expression in embryonic development

We have shown recently that *Danio rerio* has, like other bony fishes, *Agr* genes of two sub-families: *Ag1* (*DAg1*), that disappeared in higher vertebrates, and *Agr2* (*DAgr2*)^5^. The expression of one of these genes, *DAgr2*, has been already studied (Shih et al., 2007). As far as we know, however, there are no reports describing the expression of *DAg1*. Therefore, we have analyzed the expression pattern of this gene in the *Danio rerio* embryonic development.

**Figure 1:**
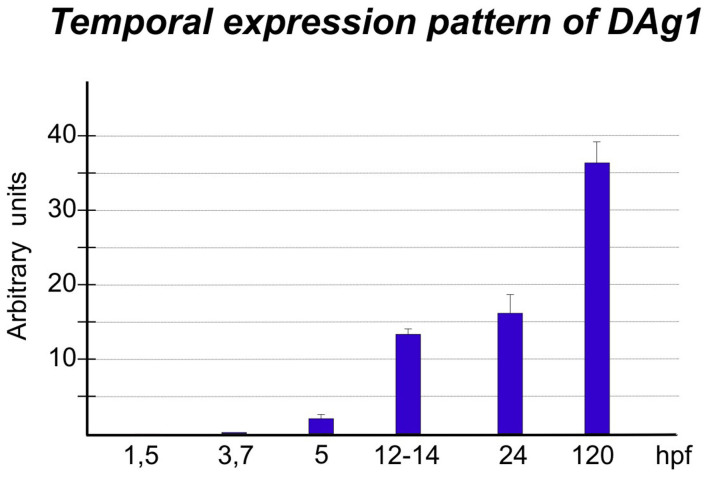
Temporal pattern of *DAg1* expression in *Danio rerio* embryogenesis. QRT-PCR of total RNA from embryos at the indicated stages with primers to cDNAs of *DAg1*. The data were normalized by using results of qRT-PCR with primers to transcripts of two housekeeping genes: *Ef1-alpha* and *ODC* (see Materials and Methods for details).

First of all, we have studied the temporal expression pattern of *DAg1* by making qRT-PCR with specific primers at the following development stages: stage 8–16-cells, (1,5 hours post fertilization, cleavage period), oblong stage (3,7 hpf, blastula period), 40–50% epiboly stage (5 hpf, gastrula period), 6–12 somite stage (12–14 hpf, segmentation period), prim-5 stage (24 hpf, pharyngula period) and 120 hpf (larval period). As a result, we have found out that *DAg1* expression starts during the blastula to gastrula transition period and then gradually increases till larval period (Fig. 1).

**Figure 2:**
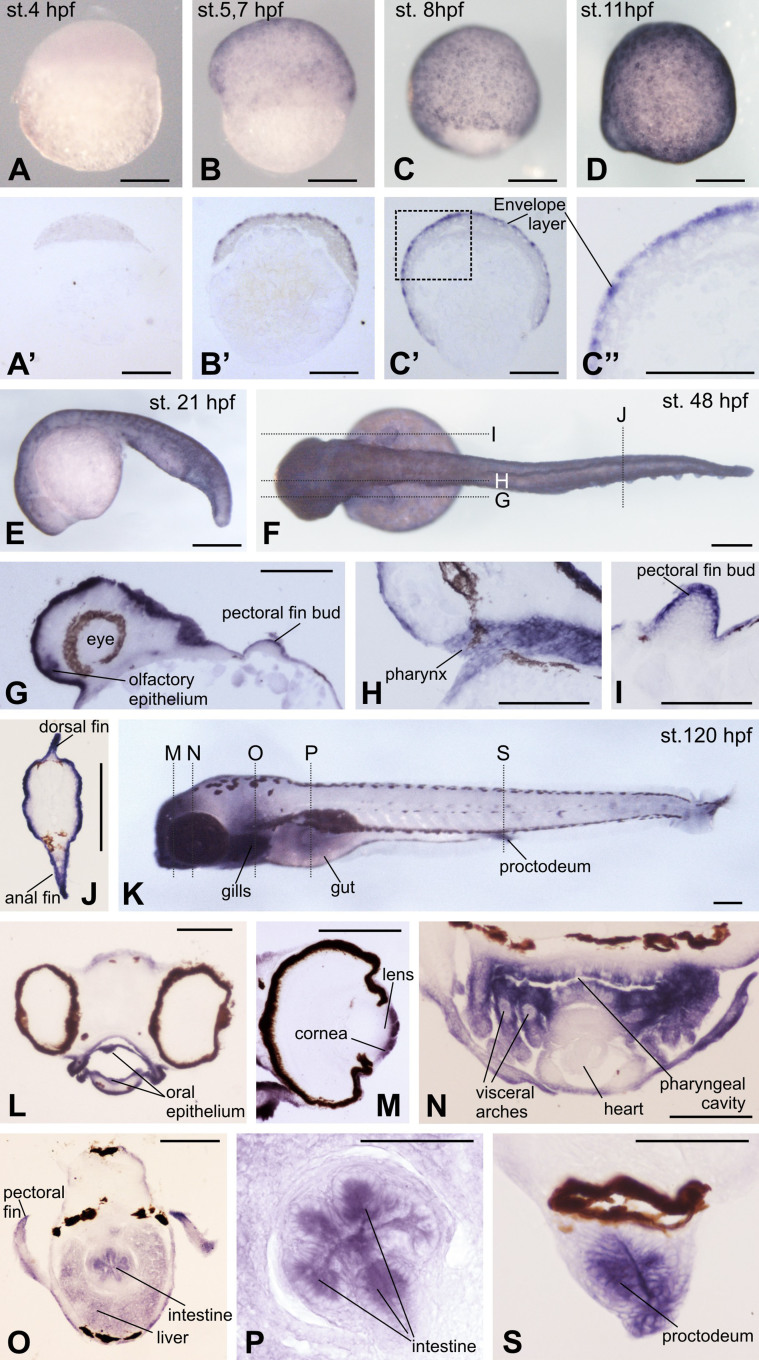
Spatial expression pattern of *DAg1* in *Danio rerio* embryos as revealed by in situ hybridization. (A–D and E). Up to 21 hpf, *DAg1* is expressed only in the superficial enveloping layer. (A′–C′). Histological sections of embryos shown on A–C. (C″). Enlarged fragment framed on C′ by dotted line. (F–J). Dorsal view of the embryo at hatching stage (F) and histological sections (G–J) of sibling embryos corresponding to the planes indicated by dotted lines on F. (K–S). Left side view of embryo at 5 days stage (K) and histological sections (L–S)of sibling embryos corresponding to the planes indicated by dotted lines on K. Scale bar everywhere is 200 μm.

To study the spatial expression pattern of *DAg1* during *Danio rerio* embryogenesis, we have used the method of the whole-mount in situ hybridization with the digoxygenin-labeled anti-sense RNA probe prepared on the base of the previously cloned cDNA template (see Materials and Methods). As a result, we have confirmed the data obtained by qRT-PCR by revealing a uniform expression of *DAg1* in all cells of the surface layer of the embryonic ectoderm at stage 30–40% of epiboly (Fig. 2A and B) and then by observing progressive increase of the signal in these cells at later stages (Fig. 2A–F). To reveal possible sites of *DAg1* expression in the inner structures, these embryos were embedded into paraplast and sectioned onto histological slices. This has revealed that at least before the pharyngula period, the expression of *DAg1* could be seen exclusively in the outermost enveloping layer of the embryo (Fig. 2A′–C″). At hatching and larval period, however, the expression was revealed, besides the surface epithelium, in the pectoral fin buds, olfactory epithelium of the nasal pit, in the gill epithelium and in the epithelial cells lining the pharynx, the intestine and the proctodeum (Fig. 2G–J and K–S). Also, *DAg1* is expressed at this period in such derivatives of the embryonic ectodermal epithelia as eye cornea (Fig. 2M).

To control specificity of in situ hybridization with *DAg1* probe, we arranged two types of the control experiments. First, we made in situ hybridization on embryos preliminary cut in halves. As a result, no additional sites of expression, besides those observed during hybridization of the whole embryos, were revealed (see Supplementary Fig. S1A). Second, we performed in situ hybridization with sense probe. In this case, no signal was seen at all (see Supplementary Fig. S1B and C). Thus, both these controls confirm specificity of the revealed pattern of *DAg1* expression.

### Expression of *DAg1* and *DAgr2* during fins regeneration

Due to its ability to easily repair amputated fins, *Danio rerio* is a suitable model for the investigation of the regeneration mechanisms. Given this, and taking in mind recently revealed involvement of the amphibians *Agr* genes in processes of the tail and the hindlimb bud regeneration, we have studied the expression of *DAg1* during regeneration of different fins of the adult fishes. In addition, the expression of another fish representative of *Agr* family, *DAgr2*, has been analyzed, since it was not yet investigated during regeneration elsewhere. As an internal control, we have also tested in these experiments the expression of two regeneration markers, *Igf2b* and *Fgf20a*, which were shown before to be strongly activated after the caudal fin amputation^11^,^12^. To be able to compare the target genes expression levels in different experiments, the geometric mean of expression levels of two reference housekeeping genes, *ornithine decarboxylase* (*ODC*) *and elongation factor 1alpa* (*EF-1alpha*), was determined in each of experiments for normalization of the data obtained.

**Figure 3:**
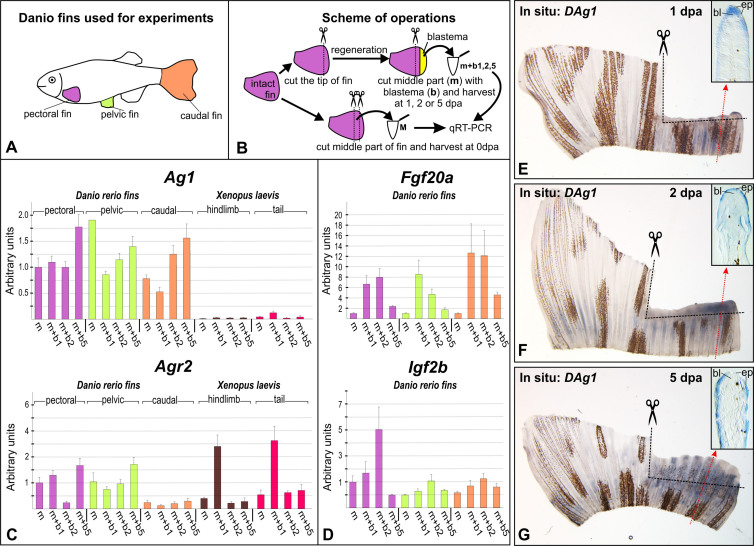
Analysis of *Ag1*, *Agr2, Fgf20a and IGF2b* expression in the regenerating fins. (A and B). Schemes of experiments. Tissue pieces of hindlimbs and tails of stage 52 *Xenopus laevis* tadpoles were collected as was previously described^5^. Drawings for these figures were done by MBT and AGZ. (C and D). QRT-PCR analysis of *Ag1* and *Agr2* (C) and *Fgf20a* and *Igf2b* (D) expression in the intact and regenerating of fins of adult *Danio rerio* and in hindlimbs and tails of stage 52 *Xenopus laevis* tadpoles. All graphs represent means of triplicate experiments. Bars indicate standard deviations. The geometric mean of expression of *Danio rerio* and *Xenopus laevis* *ornithine decarboxylase* (ODC) and *elongation factor 1alpa* (*EF-1alpha*) was used for normalization of experimental values (see Materials and Methods for details). The value of normalized PCR signal in "m" sample taken from pectoral fin was taken as an arbitrary unit in each series. (E–G). In situ hybridization of regenerating caudal fins with the probe to *DAg1* at 1, 2 and 5 dpa. Photos on insets demonstrate histological sections of regenerating fins at levels whose approximate positions are indicated by red dotted arrowed lines. Black dashed lines indicate places of amputations. Abbreviations: bl - blastema, ep - epidermis.

A common feature of the post-amputation expression dynamics revealed for *DAg1* and *DAgr2* in the distal parts of stumps of all types of the tested fins was some drop of their expression levels at the first day post-amputation 1 dpa (Fig. 3C, m + b1). Meanwhile, the expression of both these genes has started to be gradually increased at later stages, such that by 5 dpa it has exceeded the expression levels in the intact fins (Fig. 3C, m and m + b5).

By contrast, the expression of *Fgf20a* and *Igf2b* sharply increased already at 1 dpa and then decreased by 5 dpa to the intact level (Fig. 3D). Such temporal expression patterns of these two genes correspond well to the literature data, thus validating other results obtained in our experiments.

The revealed dynamics of *Agr* expression in the amputated *Danio* fins obviously differs from that recently revealed for same genes during regeneration of the *Xenopus* tadpoles tails and hindlimb buds^5^. By contrast to the initially high concentrations of *Agr* transcripts in *Danio* fins and dropping of these concentrations at 1 dpa, we observed low concentrations of *XAg1* and *XAgr2* transcripts in the intact tadpoles' tails and hindlimb buds and an increase of their concentrations after amputation.

If to assume that concentrations of housekeeping *EF-1*alpha and *ODC* transcripts are approximately similar in the fish fins and in the body appendages of the *Xenopus* tadpoles, then, knowing PCR effectiveness of all the compared templates, one may compare the expression levels of both *Agr* transcripts in these species. By this way, we have found out that while *DAgr2* concentration in the intact *Danio* fins was in average in 3–4 times higher than the concentration of its ortholog *Xagr2* in the *Xenopus* body appendages, the concentration of *DAg1* exceeded that of *XAg1* more than in 15–20 times (see PCR effectiveness for the *Danio rerio* and *Xenopus laevis* *Ag1*, *Agr2*, *EF-1alfa* and *ODC* in Materials and Methods and in Table 1). Importantly, the concentration of *DAg1* transcripts after its dropping at 1 dpa in the amputated fins still remained higher than the increased concentration of *XAg1* in the amputated body appendages of the *Xenopus* tadpoles (Fig. 3C). Thus, if to suppose that the increased concentration of *XAg1* transcripts in the amputated tadpole appendages at 1 dpa is critical to ensure necessary level of the activity of this protein for the regeneration process, then the concentration of *DAg1* transcripts detected at 1 dpa in the amputated fins could be also sufficient for the same function.

The results of qRT-PCR experiments were confirmed by in situ hybridization with *DAg1* probe (Fig. 3E–G). Interestingly, at 1 and 2 dpa the revealed signal was higher in more rapidly growing lateral parts of the amputated fins (Fig. 3E–F). As we have shown by histological sectioning, *DAg1* is expressed in these regenerating parts of fins mainly in epidermis and in cells of the regenerating blastema, located just beneath the wound epithelium. Noteworthy, the expression of *DAg1* in the blastema cells was especially evident in the 1 dpa fins. In general, this expression pattern of *DAg1* very resembles the expression of its orthologs, *Xag1/2*, which we observed earlier in the regenerating tails and limb buds of the *Xenopus laevis* tadpoles^5^.

### *DAg1* is essential for regeneration of the caudal fin

To test directly if *DAg1* is actually involved in the mechanism of the fin regeneration, we sought to inhibit translation of its mRNA by injecting into the stumps of the amputated caudal fins of antisense Vivo-morpholino oligonucleotides (*Vivo-MO*). The efficiency of this MO was tested and confirmed in experiments in which synthetic mRNA encoding for the Myc-tagged DAg1 was injected into the *Xenopus laevis* embryos either in a mixture with *DAg1* VivoMO or with *control-misDAg1* VivoMO (see Supplementary Fig. S2).

**Figure 4:**
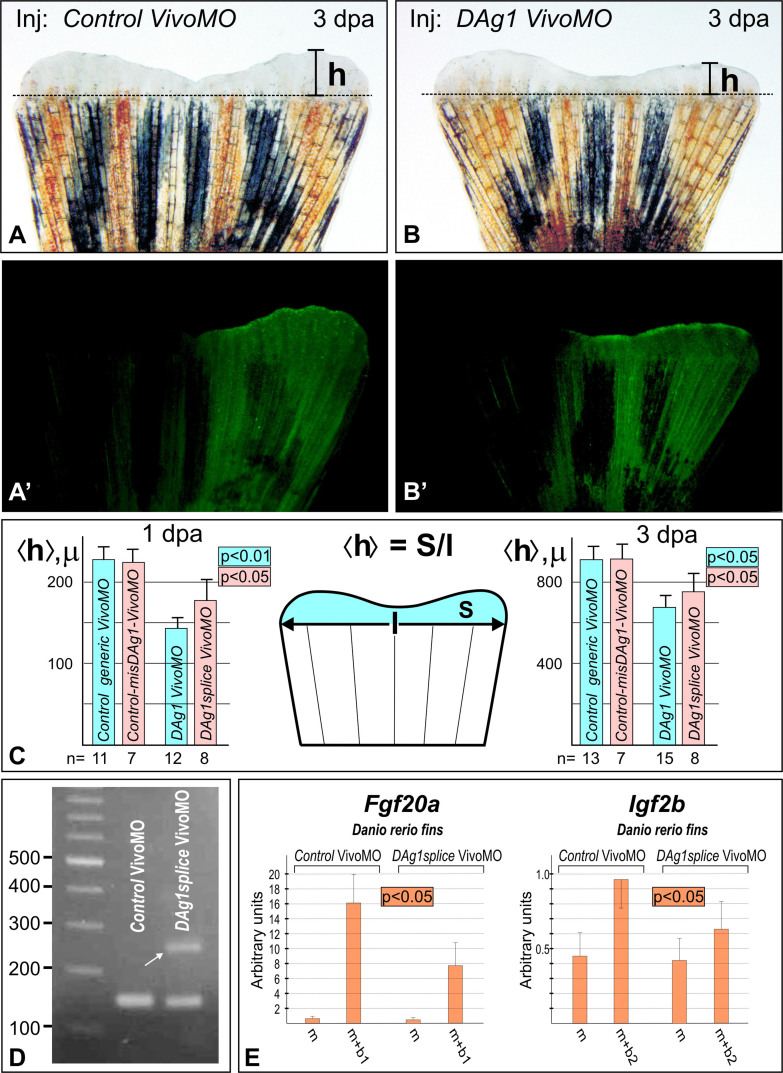
Blocking of *DAg1* mRNA translation by Vivo-morpholino injections results in retardation of the caudal fin regeneration. (A and A'). Caudal fin injected by the *control-generic* Vivo-morpholino mixed with FLD in the right side. No retardation of regeneration is seen on the injected side. Dashed line indicates the level of amputation. Scale bar 1 mm. (B and B'). Caudal fin injected by the *DAg1* Vivo-morpholino mixed with FLD in the right side. Note retardation of regeneration of the injected side. Scale bar 1 mm. (C). Quantification of the mean height of the regenerating part of caudal fins injected by *control-generic* Vivo MO or *DAg1* Vivo MO at 1 dpa (left) and 3 dpa (right). Numbers of the injected fins are indicated by n. The schema in the middle demonstrates how mean height <h> was calculated. Drawing on this figure was done by AGZ. (D). RT-PCR analysis of *DAg1* transcripts in 1 dpa regenerating caudal fins injected with either *control-generic* Vivo MO or *DAg1splice* Vivo MO. White arrow indicates additional band generated by *DAg1* un-spliced transcript. (E). QRT-PCR analysis of *Fgf20a* and *Igf2b* expression in the regenerating caudal fin at 1 dpa and 2 dpa respectively (at these days the expression levels of *Fgf20a* and *Igf2b* reach maximal values, see Fig. 3D) maximal expression levels of these genes injected with either *control-generic* Vivo MO or *DAg1splice* Vivo MO. For scheme of experiments and abbreviations see picture D and legends on Fig. 3).

As a result of injections of the amputated fins with *DAg1Vivo* MO, we observed statistically significant retardation of the fin regeneration on the injected side (Fig. 4 and Supplementary Fig. S3). Importantly, no retardation of regeneration was seen in fins injected with any of two types of the control MO: *control*-*generic* VivoMO or *control*-*misDAg1* VivoMO ( Fig. 4 A and C, Supplementary Fig. S3A and C).

To test specificity of *DAg1 Vivo-MO* effect, we used another *Vivo-MO*, designed to a region that included donor splicing site at the border of exon 2 and intron 2–3 of *DAg1* gene (Supplementary Fig. S4). Blocking of this splicing site should potentially result in the translation frame-shifting, accompanied by an extension of RNA template on additional 104 nucleotides in comparison with the normal spliced mRNA. When this *DAg1splice Vivo-MO* was injected, we did observe a retardation of the caudal fin regeneration, while less expressed than in case of *DAg1 Vivo-MO* injections. Importantly, the longer form of *DAg1* transcript was detected by RT-PCR when 2 dpa tissue samples of regenerating fins were analyzed, whereas no such form of *DAg1* transcripts was revealed in the control fins (Fig. 4D). Obviously, this result confirms that *DAg1splice Vivo-MO* indeed penetrate even into the nucleus and block splicing of *DAg1* transcripts. However, this blocking was not full, probably because relatively small amount of MO was able to pass, beginning from the intercellular space, through all the cytoplasm and to reach its targets within cell nuclei.

To further test specificity of *DAg1splice Vivo-MO* effects, we analyzed by qRT-PCR the expression of two regeneration markers, *Fgf20* and *IGF*, in the control and *DAg1splice Vivo-MO*-injected regenerating fins. As a result, a moderate but statistically significant inhibition of the expression of both genes by *DAg1splice Vivo-MO* was observed (Fig. 4E).

**Figure 5:**
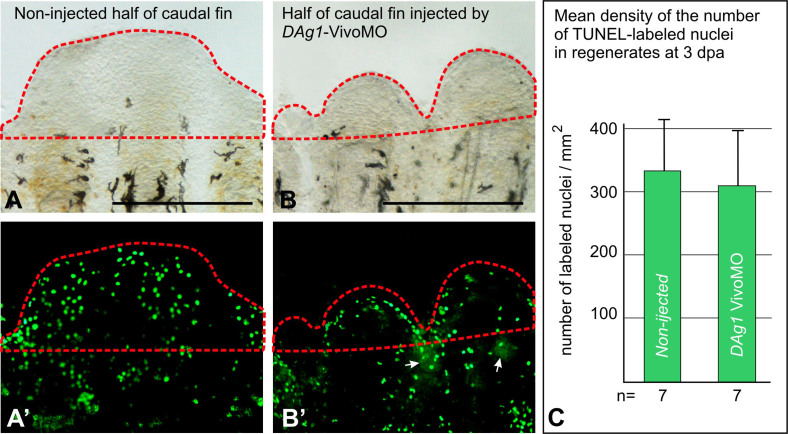
TUNEL assay of apoptosis in the regenerating caudal fins injected with Vivo MO. (A and A′). Revealing by TUNEL assay of apoptotic nuclei in regenerating part (framed by red dashed line) of the non-injected side of the caudal fin at 3 dpa. Scale bar 500 μm. (B and B′). Revealing by TUNEL assay of apoptotic nuclei in regenerating part (framed by red dashed line) of the side of caudal fin injected by *DAg1*Vivo MO at 3 dpa. White arrows indicate traces of FLD co-injected with MO. Scale bar 500 μm. (C). Quantification of TUNEL-labeled nuclei in the regenerating parts (regenerates) of caudal fins non-injected and injected by *DAg1* Vivo MO.

Finally, to verify if the retardation of regeneration observed in the case of *DAg1-Vivo*MO could be caused by cell death, which is a non-specific effect seen with some MOs, we investigated cell apoptosis in regenerating fins injected with *DAg1* VivoMO by means of TUNEL assay. As a result, no statistically significant difference in average concentration of apoptotic cells in regenerates was revealed between normally regenerating non-injected parts of the fins and their retarded parts that were injected with *DAg1* VivoMO (Fig. 5). Thus, we concluded that the observed effect of the retardation of the fin regeneration was not the result of cell death.

The retardation of regeneration of the amputated fins injected by *DAg1 Vivo-MO*, compared with the fins injected by control *Vivo-MOs*, was observed within 3–5 days after injection, during which the fins were repeatedly injected by *Vivo-MO* solution. After the cessation of repeated injections, the difference with the control fins gradually disappeared within a few days. In sum, these data confirm an essential role of *DAg1* for the fin regeneration.

## Discussion

In the present work, we have cloned for the first time cDNA of the *Danio rerio* ortholog of the *Xenopus* *XAg1* gene, *DAg1*, studied its expression pattern in early embryogenesis and demonstrated its role in the process of the caudal fin regeneration.

As we have revealed, the expression of this gene is localized in the embryonic epitheliums, thus, resembling in that the expression of *DAg1* ortholog in *Xenopus laevis*, which is also activated for the first time in the ectodermal epithelium beginning from the late blastula stage^4^,^13^. However, in contrast to its *Xenopus* counterpart, we were unable to reveal any signs of the anterior to posterior gradient of *DAg1* expression. Instead, this gene is expressed quite uniformly in all cells of the superficial ectodermal layer of the embryo till larval stages. Similarly to *XAg1* in Xenopus, *DAg1* is also expressed at the hatching stage in cells of other tissues of epithelium origin, including, olfactory pits, gills, pharynx, intestine, proctodeum and cornea.

In the last decade, more and more data is accumulated, which demonstrate involvement of Agr proteins in regulation of many processes, including cell proliferation, cell differentiation and cancerogenesis^6^,^8^,^14^. As we have shown recently, the *Xenopus* ortholog of *DAg1* is intensively expressed in the anterior non-neural subdomain of the ectodermal epithelium, adjacent to cells of the telencephalic primordium located at the anterior border of the neural plate, and regulates development of the telencephalon by promoting the Fgf8 signaling^4^. A similar location of the surface ectoderm cells expressing *DAg1*, adjacent to cells of the presumptive telencephalon, was revealed now in *Danio* embryos. In connection with this, it would be interesting to verify in the future experiments whether *DAg1* can also regulate the telencephalic development in *Danio*, like its ortholog *XAg1* does this in *Xenopus*.

As we have demonstrated in the present work, the expression of *DAg1*, like the expression of its *Xenopus* ortholog *Xag1*, increases after amputation of the body appendages (fins). Unlike the hindlimbs and tails of the *Xenopus* tadpoles, however, a significantly higher expression level of *DAg1* is revealed already in the intact fins before amputation. Given our data demonstrating an essential role of DAg1 for the regeneration process, one may hypothesized that such a high basal level of its expression is necessary to ensure timely participation of DAg1 in the repairing of the adult fish fins, which might be more frequent and critical for survival of the population than damage of limbs and tails of the *Xenopus* tadpoles.

It was shown that the expression of one of Agr proteins, Agr2, is activated after amputation of the newt limb, and Agr2 functioning is critical for the process of the limb regeneration^3^,^7^. In consistent with this, we have revealed recently the activation of expression of both *Ag1* and *Agr2* after amputation of the tail or hindlimb bud in the *Xenopus laevis* tadpoles^5^. Importantly, we have also shown in the cited work that *Ag1* had disappeared somewhere during evolutionary transition from the amphibians to higher vertebrates. Given all these data, along with the results of the present work which demonstrate an essential role of *DAg1* for the fin regeneration in *Danio*, one may hypothesizes that disappearance of *Ag1* family genes in evolution could be one of the critical events, which resulted in a reduced regenerative capacity observed in extant higher vertebrates.

## Methods

Animal experiments were performed in accordance with guidelines approved by the Shemyakin-Ovchinnikov Institute of Bioorganic Chemistry (Moscow, Russia) Animal Committee and handled in accordance with the Animals (Scientific Procedures) Act 1986 and Helsinki Declaration.

### Cloning of DAg1 cDNA and dig-probe synthesis

For generating the template for synthesis of the *DAg1* probe, DNA fragment corresponding to the protein coding region of DAg1 cDNA was obtained by RT-PCR from the first strand obtained from the total zebrafish embryos (48 hpf ) RNA with the following primers:

DAg1 forward: 5′-ATGAATTCTTATGAAATTCTGAAGACA,

DAg1 reverse: 5′-GAGTCGACGATCAGAGTTCAGTCTGC.

The obtained DNA fragment was cloned in pGEM-T (Promega) vector and correct clone was selected as a result of sequencing of five random clones. The resulting Dig-labeled RNA antisense probe for the whole-mount in situ hybridization was synthesized by T7 polymerase (mMessageMachine) from the PCR-product obtained with DAg1 forward and M13 reverse primers from *DAg1pGEM-T* plasmid construct.

To construct templates for testing the efficiency of *DAg1 VivoMo, DAg1* cDNA was obtained by PCR with the following primers (restriction sites of BamHI and NcoI are underlined, start- and stop-codones are in frames):

Forward-DAg1- 5'-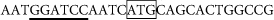 and Reverse-DAg1- 5'-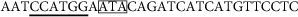.

The obtained cDNA was sub-cloned into pCS2-Myc-tag by BamHI and NcoI, upstream and in frame with the last Myc-tag. Capped synthetic mRNA encoding for DAg1-Myc was synthesized with SP6 Message Machine Kit (Ambion) using the obtained plasmids cut by NotI.

### Animals handling, samples fixation, whole-mount in situ hybridization and hystological sections

*Danio rerio* embryos were staged according to standard staging table (Westerfield, 2000).

In regeneration experiments adult fishes were anesthetized in 0.1% tricaine (Sigma, Aldrich). *Xenopus laevis* tadpoles were obtained as we described previously^5^. Fin amputations were performed in adult fishes with razor blades or with microscissor. The latter was used when a half of fin should be amputated. Animals were allowed to regenerate for various time periods at 29,5°C and the fins were cut off again and fixed for qRT-PCR or in situ hybridization. Tails and hind limb buds of the *Xenopus laevis* tadpoles at stage 52 were amputated by microscissors and were further processed for RT-PCR analysis at different days postamputation as we previously described^5^. For in situ hybridization, *Danio rerio* embryos or caudal fins were fixed in MEMFA (3.7% paraformaldehyde, 2мМ EGTA, 1мМ MgSO_4_х7H_2_O, 0.1мМ MOPS, pH 7.4) during 24 hours, dehydrated by ethanol and processed for in situ hybridization. The in situ hybridization was performed as described^5^. For histological sectioning, embryos or fins were embedded in paraplast and cut into 15 μm sections. To control the penetrability of embryos for *DAg1* probe, some embryos were cut as previously described before the hybridization procedure by a microknife^4^. Also, to control specificity, some embryos were hybridized with sense probe to *DAg1*.

### QRT-PCR

The total RNA from whole embryos or amputated pieces of fins (see scheme of operation on Fig 3A and B) was extracted by RNA isolation KIT (MASHEREY-NAGEL) according to the manufacturer's protocol. For each type of samples shown on Fig. 3B, three replicates, each containing RNA from five amputated pieces of the same type, were prepared. The concentration of the extracted RNA was measured by means of Qubit® fluorometer (Invitrogene), while RNA integrity was checked visually by gel electrophoresis. 250 ng of total RNA extracted from each sample was reverse transcribed in 20 μl of final volume by M-MLV reverse transcriptase (Promega) in presence of 10 pmol of oligo-dT primer (Evrogen), according to the manufacturer's guidelines (Promega) (+RT sample). In parallel, same reaction was assembled in each case without adding of M-MLV reverse transcriptase (-RT control). For qPCR reaction, which was performed on a ANK-32 (Syntol), 2 μl of + RT and -RT solutions of each type were mixed in parallel tubes with qPCRmix-HS SYBR (x5, Evrogen), corresponding primers (5 pmol each) and milli-Q water till the final volume 25 μl. A standard 40-cycle program with hot start was used; the annealing temperature was 59°C, elongation −72°C and melting 95°C, all lasted for 25 seconds. The PCR data were imported into Microsoft Excel and analyzed by using the ΔΔ*C*_t_ method. The geometric mean of expression of two reference housekeeping genes: *ornithine decarboxylase* (ODC)^15^ and *elongation factor 1alpa* (*EF-1alpha*) was used for normalization of the target genes expression levels. The following pairs of primers for *Danio rerio* genes were used:

*ODC* forward 5′- CTCCACCTTCAATGGCTTCCAG;

*ODC* reverse 5′- AGTGGGATGGCACGTTTCCAG;

*EF-1alpha* forward 5′- AAGAACGTGTCAGTCAAGGACAT;

*EF-1alpha* reverse 5′ - CGTAACCCTGAGAGATCTGACCA;

*Fgf20a* forward 5′- AAGGGCGAACTGTACGGAT;

*Fgf20a* reverse 5′- TTGAGGGCTACATAATAAT;

*Igf2b* forward 5′ - GCAGGTCATTCCAGTGATGC;

*Igf2b* reverse 5′- TCTGAGCAGCCTTTCTTTGC;

*DAgr2* forward 5′- AACCACAGAGCGTGTCAGT;

*DAgr2* reverse 5′- ACAGTGCTAATGCTTTCATTG;

*DAg1* forward 5′- CACTGGCCGCTCTGTATACTT;

*DAg1* reverse 5′- CTCTTGAGAGAGTTTGGACTGT;

*DAg1* reverse-2 primer for revealing by qRT-PCR of the unspliced form of *DAg1* transcript in experiments with *DAg1splice* VivoMO and used in pair with *DAg1* forward primer 5′-AGCCAGTCCCTCCTCATACG.

Primers for the *Xenopus laevis* *Xag2*, *Xagr2*, *ODC* and *EF-1alpha* were the same as in^5^.

PCR efficiency (*PE*) for Xag1, DAg1, Xagr2, Dagr2, XEFalfa, DEFalfa, XODC and DODC were calculated by making qPCR form progressive dilutions of the 1st strand of cDNA obtained on the base of total RNA samples extracted from *Xenopus laevis* and *Danio rerio* midneurula stage embryos, building standard curves and using the on-line qPCR efficiency calculator (http://www.thermoscientificbio.com/webtools/qpcrefficiency). As a result, the following *PE* values were obtained (see Table 1). As one may see, *PEs* for orthologous *Agrs* as well as for orthologous housekeeping genes appear to be very similar. This fact justifies the comparison of concentrations of the *Xenopus* and *Danio* *Agrs* templates normalized in relation to concentrations of housekeeping templates.

For visualization of additional band generated by un-spliced DAg1 nuclear transcript in experiments with *DAg1splice* VivoMO, the PCR mixture after completion of qRT-PCR was resolved in 2% agarose gel and photographed.

### Vivo-morpholino injections and fin length measurements

To test effects of *DAg1* functioning down-regulation during fin regeneration, the 0,4 mM solutions of vivo-porter morpholino oligonucleotides (Gene-Tools) mixed with the tracer FLD (fluorescein lysinated dextran, Invitrogen, 40 kDa, 5 μg/μl)) were used for injections into caudal fin tissues. The following Vivo-MOs were used:

*DAg1* VivoMO 5′ ACAGAGCGGCCAGTGCTGCATGATT;

*DAg1splice* VivoMO 5' AATTTGCTGATACCTCTTGAGAGAG (sequence complementary to the 2nd exon is underlined; for genomic sequence see Ensembl Acc.: ENSDARG00000060682);

*control-generic* VivoMO 5′ TCTgTggATgTCTTgCTCTTCCAgg;

*control-misDAg1* VivoMO 5' ACAGATCGGCAAGTTCTGCATTATT.

The day before amputation, caudal fins of fishes anesthetized by Tricain were pre-injected with the use of Eppendorf FemtoJet microinjector by Vivo-MOs mixed with FLD into the area between bony rays along the line of the subsequent plane of amputation (4 nl per one injection, 12–15 injections per fin) in order to pre-inhibit the strong basal *DAg1* expression. Amputations were performed with razor blades along the FLD-marked line under the fluorescent stereomicroscope Leica M205. Immediately after amputation, stumps of the fins were injected with vivo-MOs once again, and then injections were repeated at 24 hpa and 48 hpa. The fins were photographed by using Leica M205 stereomicroscope and Leica camera (DC 400F). To calculate the mean of the regenerate length of the caudal fins (from the amputation plane to the distal tip of the fin) injected by the control or *anti-DAg1* vivo-MOs, the following formula was used: S/L, where L was the fin width and S was the square of the regenerate, both measured by using ImageJ software (http://rsb.info.nih.gov/ij). Statistical significance was determined with the Student's test and the significance was set at P < 0,01 or P < 0,05.

To test efficiency and specificity of *DAg1* Vivo MO, *DAg1-Myc* mRNA (see above) was injected into animal pole of 4-cells *Xenopus laevis* embryos (100 pg/blastomere) either alone or in a mixture with *mi*s-*DAg1* VivoMo MO or *DAg1* VivoMo (6–8 nl of 0,2 mM water solution). The injected embryos were collected at the gastrula stage and analyzed for the presence of DAg1-Myc by Western blotting with Sigma Anti-Myc antibody (cat. # M 4439) as described previously^16^. As a result, no inhibition of *DAg1-Myc* mRNA translation was observed in embryos co-injected with this mRNA and mis-*DAg1* VivoMo MO. By contrast, a strong suppression of *DAg1-Myc* mRNA translation was observed in embryos co-injected with this mRNA and *DAg1-*Vivo MO (Fig. S2). These results confirm the efficiency and specificity of the latter MO.

### TUNEL assay

For this assay, caudal amputated caudal fins were injected in one side as described above. The only difference is that *DAg1-*Vivo MO was mixed with a decreased amount of FLD (1 μg/μl). This trick allowed us to mark the injected side and simultaneously to reveal FITC-labeled nuclei on the low diffuse signal emitted by FLD. TUNEL assay was done on the whole-mounted caudal fins fixed in MEMFA. To reveal apoptotic cells, DeadEnd™ Fluorometric TUNEL System (Promega) was used in accordance with the manufacturer's recommendations. The number of labeled nuclei and their density (number of labeled nuclei/mm^2^ of the planar projection of regenerate) were calculated in the MO-injected and non-injected part of each fin by using ImageJ software.

## Author Contributions

A.S.I. and I.M.S. performed cloning, qRT-PCR, in situ hybridization and M.O. injections. G.V.E. arranged and performed TUNEL experiments. A.A.M. collected and fixed embryos. M.B.T. and A.G.Z. designed experiments, drew and composed figures and wrote the manuscript.

## Supplementary Material

Supplementary Information Supplementary Information

## Figures and Tables

**Table 1:** PCR efficiency of analyzed cDNA

	*Xag1*	*DAg1*	*Xagr2*	*Dagr2*	*XEFalfa*	*DEFalfa*	*XODC*	*DODC*
PCR Efficiency	82%	83%	82%	80%	90%	92%	93%	90%
