# Supplementary material for: The secreted factor Ag1 missing in higher vertebrates regulates fins regeneration in Danio rerio
Source: Sci Rep. 2015 Jan 29;5:8123. doi: 10.1038/srep08123 (PMC4309956; doi:10.1038/srep08123)

**The secreted factor Ag1 missing in higher vertebrates regulates fins regeneration in *Danio rerio*.**

**Anastasiya S. Ivanova^+1^, Igor N. Shandarin^+1^, Galina V. Ermakova^1^, Andrey A. Minin^2^, Maria B. Tereshina^1^*, Andrey G. Zaraisky^1^***

**^1^**Shemyakin-Ovchinnikov Institute of Bioorganic Chemistry, Russian Academy of Sciences, Moscow, 117997, Russia.

**^2^**Koltzov Institute of Developmental Biology, Russian Academy of Sciences,

Moscow, 117997, Russia.

**Supplementary Figure Legends**

**Supplementary Figure S1. Control in situ hybridization.**

**A.** In situ with antisense *DAg1* probe on the 11 hpf embryo cut sagittally before hybridization. *DAg1* expression is seen only in the superficial layer of cells, but not in cells of deeper layers. This confirms that the in situ hybridization signal, which was observed primarily in the superficial cells of embryos hybridized in whole-mount, was not the result stipulated by a poor penetration of *DAg1* probe into the depth of embryo. Anterior to the left, dorsal side up.

**B and C**. No signal was detected when embryos at 8 and 36 hpf were hybridized in whole mount with sense probe to *DAg1*. This result confirms specificity of the signal observed with antisense *DAg1* probe. On C animal pole to the top. On C anterior to the left, dorsal side up.

Scale bar everywhere is 200 μm

**Supplementary Figure S2. Testing of *DAg1 Vivo* MOs efficiency.**

**A**. *DAg1-Myc* mRNA were injected into the animal parts of two blastomeres of 4-cell *Xenopus laevis* embryos (100 pg/blastomere) either alone or in a mixture with control-*mis-Dg1* VivoMO or *Dag1* VivoMO (8 nl of 0,2 mM water solution). The injected embryos were collected at the midgastrula stage and analyzed for presence of DAg1-Myc protein by Western blotting with an anti-Myc antibody (see Materials and Methods for details).

**Supplementary Figure S3. Comparison of effects of *DAg1* and control mismatch *DAg1*MO.**

**A and A'**. Caudal fin injected by the *control-misDAg1* Vivo-MO mixed with FLD in the right side. No retardation of regeneration is seen on the injected side. Dashed line indicates the level of amputation.

**B and B'**. Caudal fin injected by the *DAg1* Vivo-morpholino mixed with FLD in the right side.

Note retardation of regeneration of the injected side.

**C**. Quantification of the mean height of the regenerating part of caudal fins injected by *control-misDAg1* Vivo-MO or *DAg1* Vivo MO at 2 dpa. Numbers of the injected fins are indicated by n. The schema in the middle demonstrates how mean height <h> was calculated.

**Supplementary Figure S4. The target site for *Vivo DAg1splice* MO in *DAg1* genomic sequence.**

Target site for *Vivo DAg1* MO is framed. Exons are in capital letters, intron is in small letters. Exon/intron borders are indicated by vertical dashed lines. Target sites for qRT-PCR primers are underlined. Start codon for protein translation is in red.

**Supplementary Figure S1**


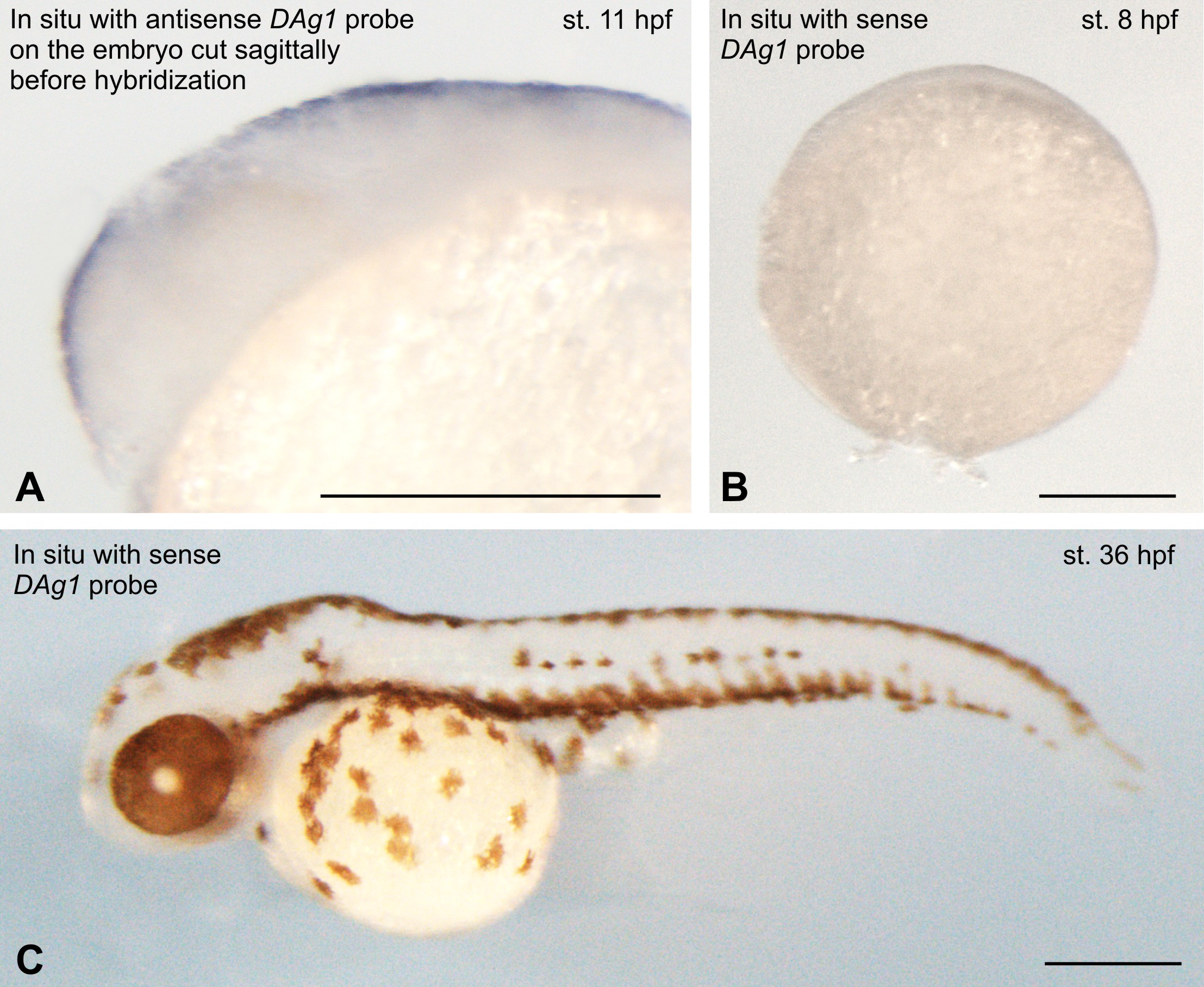


**Supplementary Figure S2**

**
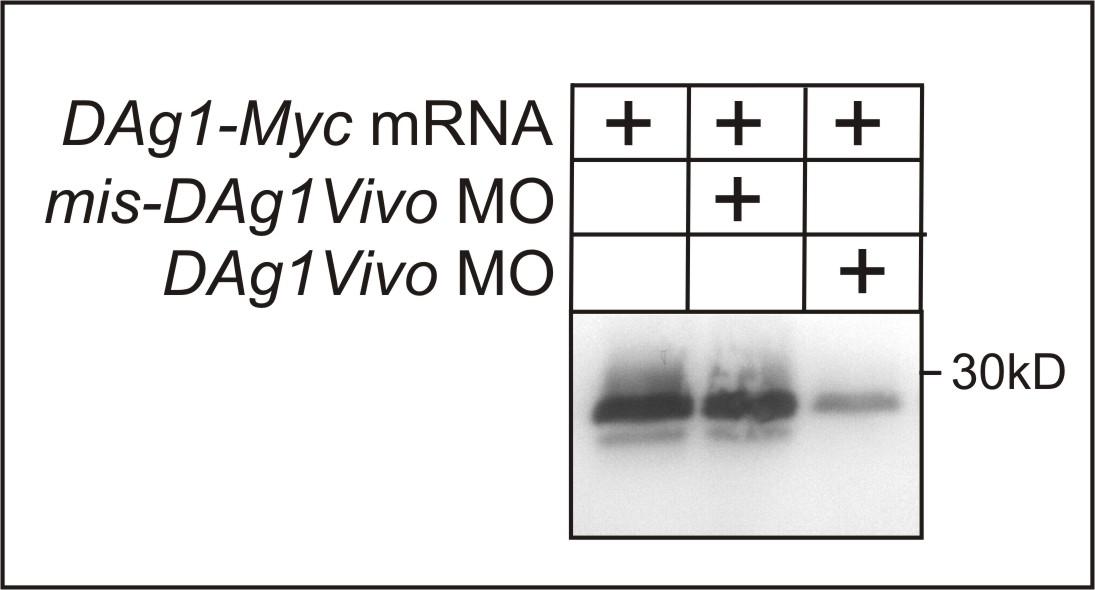
**

**Supplementary Figure S3**

**
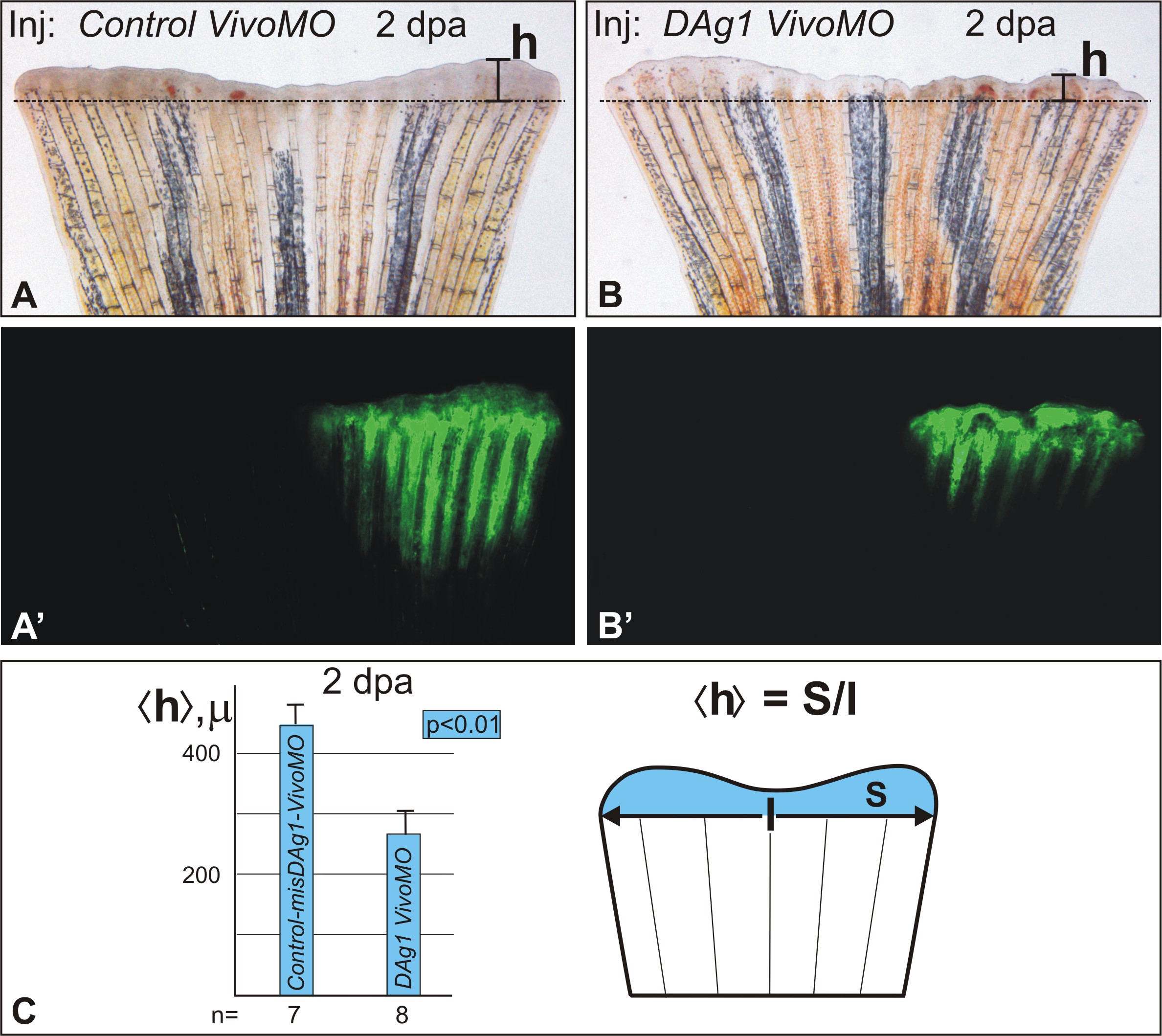
**

**Supplementary Figure S4**


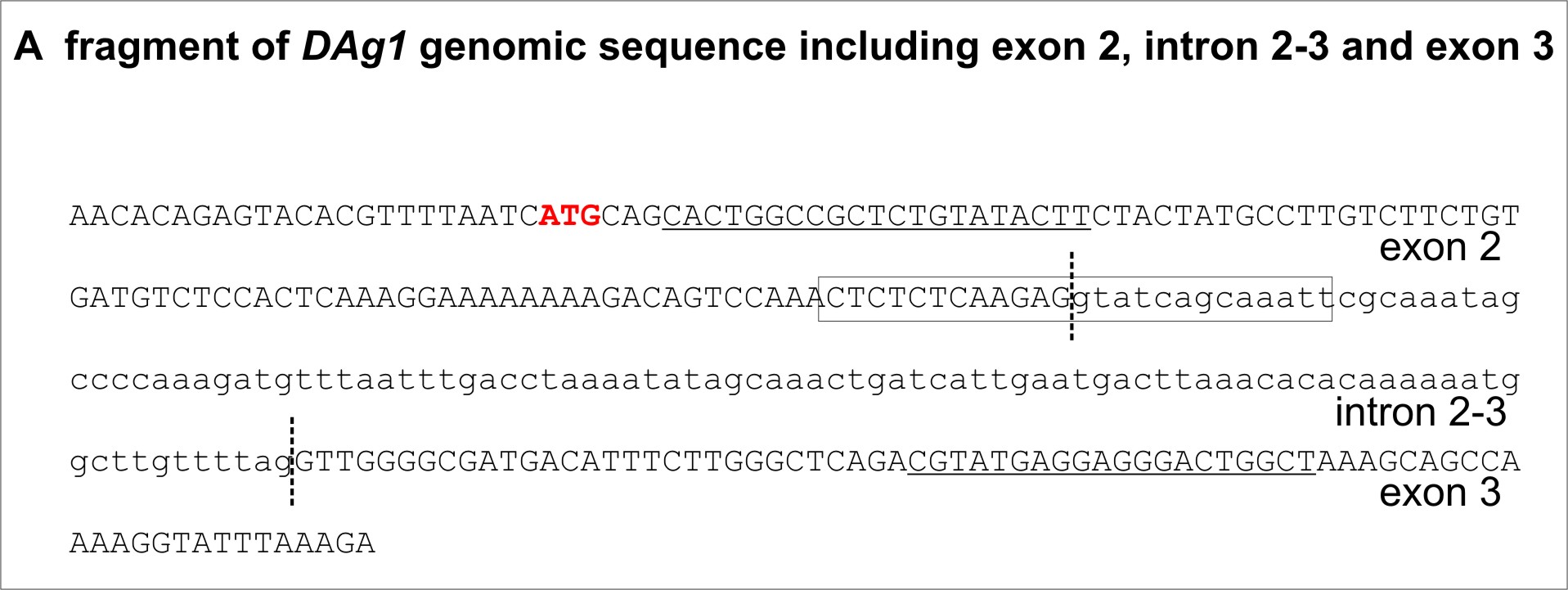

Supplement: Supplementary Information [file srep08123-s1.doc]
